# Click-to-Release: Cleavable Radioimmunoimaging with [^89^Zr]Zr-DFO-*Trans*-Cyclooctene-Trastuzumab Increases Tumor-to-Blood Ratio

**DOI:** 10.7150/thno.84865

**Published:** 2023-07-09

**Authors:** Maria Vlastara, Raffaella Rossin, Freek J.M. Hoeben, Kim E. de Roode, Milou Boswinkel, Laurens H.J. Kleijn, James Nagarajah, Mark Rijpkema, Marc S. Robillard

**Affiliations:** 1 Tagworks Pharmaceuticals, Toernooiveld 1, 6525 ED Nijmegen, The Netherlands.; 2 SyMO-Chem, Den Dolech 2, 5612 AZ Eindhoven, The Netherlands.; 3 Department of Radiology and Nuclear Medicine, Radboud University Medical Center, Geert Grooteplein Zuid 10, 6525 GA Nijmegen, The Netherlands.

**Keywords:** radioimmunoimaging, IEDDA, click-to-release, *trans*-cyclooctene, trastuzumab

## Abstract

One of the main challenges of PET imaging with ^89^Zr-labeled monoclonal antibodies (mAbs) remains the long blood circulation of the radiolabeled mAbs, leading to high background signals, decreasing image quality. To overcome this limitation, here we report the use of a bioorthogonal linker cleavage approach (click-to-release chemistry) to selectively liberate [^89^Zr]Zr-DFO from *trans*-cyclooctene-functionalized trastuzumab (TCO-Tmab) in blood, following the administration of a tetrazine compound (trigger) in BT-474 tumor-bearing mice.

**Methods:** We created a series of TCO-DFO constructs and evaluated their performance in [^89^Zr]Zr-DFO release from Tmab *in vitro* using different trigger compounds. The *in vivo* behavior of the best performing [^89^Zr]Zr-TCO-Tmab was studied in healthy mice first to determine the optimal dose of the trigger. To find the optimal time for the trigger administration, the rate of [^89^Zr]Zr-TCO-Tmab internalization was studied in BT-474 cancer cells. Finally, the trigger was administered 6 h or 24 h after [^89^Zr]Zr-TCO-Tmab- administration in tumor-bearing mice to liberate the [^89^Zr]Zr-DFO fragment. PET scans were obtained of tumor-bearing mice that received the trigger 6 h post-[^89^Zr]Zr-TCO-Tmab administration.

**Results:** The [^89^Zr]Zr-TCO-Tmab and trigger pair with the best *in vivo* properties exhibited 83% release in 50% mouse plasma. In tumor-bearing mice the tumor-blood ratios were markedly increased from 1.0 ± 0.4 to 2.3 ± 0.6 (p = 0.0057) and from 2.5 ± 0.7 to 6.6 ± 0.9 (p < 0.0001) when the trigger was administered at 6 h and 24 h post-mAb, respectively. Same day PET imaging clearly showed uptake in the tumor combined with a strongly reduced background due to the fast clearance of the released [^89^Zr]Zr-DFO-containing fragment from the circulation through the kidneys.

**Conclusions:** This is the first demonstration of the use of *trans*-cyclooctene-tetrazine click-to-release chemistry to release a radioactive chelator from a mAb in mice to increase tumor-to-blood ratios. Our results suggest that click-cleavable radioimmunoimaging may allow for substantially shorter intervals in PET imaging with full mAbs, reducing radiation doses and potentially even enabling same day imaging.

## Introduction

Monoclonal antibodies (mAbs) are great targeting agents with high uptake in tumors as a consequence of their long blood circulation 1. A variety of mAb-based biopharmaceuticals are used in cancer imaging and therapy 2. For cancer imaging using positron emission tomography (PET) and long-circulating mAbs, the radionuclide of choice is β^+^ emitting Zr-89, with its 3.3 days half-life 3,4. Some limitations to using ^89^Zr-labeled mAbs include i) the high radiation dose in excretory organs such as the liver 5,6 and ii) the need for a 4-to-7-day interval between injection of radioactivity and the PET scan to achieve sufficiently high tumor-to-blood (T/B) ratios for imaging 7,8.

A few solutions have been proposed to address these issues. Short circulating mAb fragments have been investigated, but they can exhibit suboptimal pharmacokinetics with a decreased tumor uptake due to fast renal clearance, and high kidney retention 9. Another approach is tumor pretargeting, wherein injection of a tagged mAb is followed by a small radiolabeled probe that binds the tag in the tumor or otherwise clears rapidly from blood 10. However, this method is mainly limited to the targeting of non-internalizing receptors and it typically requires the use of a clearing agent to remove the tagged mAb from circulation to the liver before probe administration, increasing the complexity of the method 11,12. While clearing agents have also been used to remove radiolabeled mAbs from blood once the desired tumor uptake has been achieved, this approach results in high radioactivity retention in liver 13-15.

**Figure 1:**
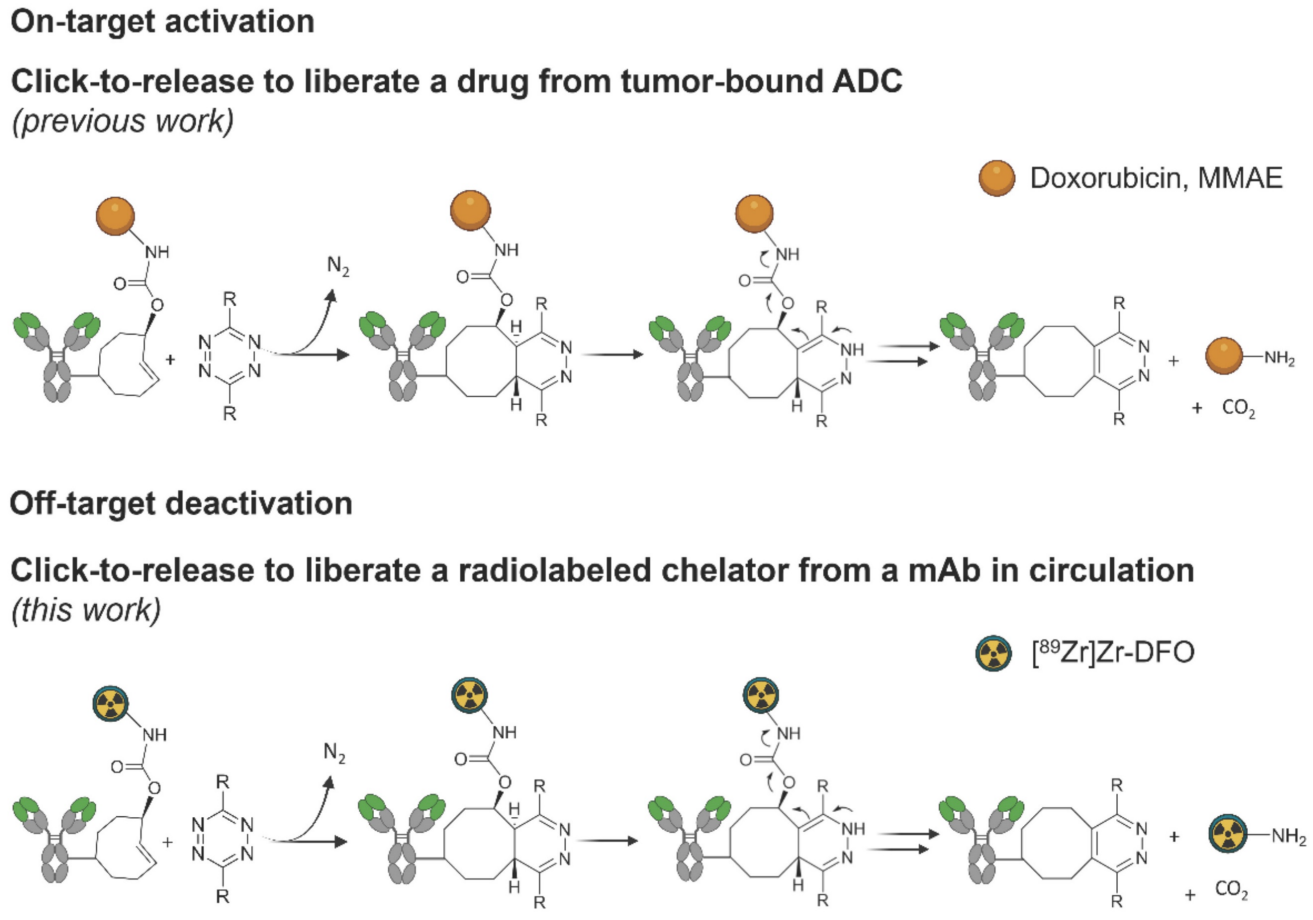
**Click-to-release concept.** Top: inverse electron-demand Diels-Alder (IEDDA) pyridazine elimination reaction between TCO and tetrazine**,** wherein the released payload is doxorubicin or MMAE (previous work) 19,20; bottom: IEDDA pyridazine elimination reaction wherein the released payload is a radioactive moiety (this work).

**Figure 2:**
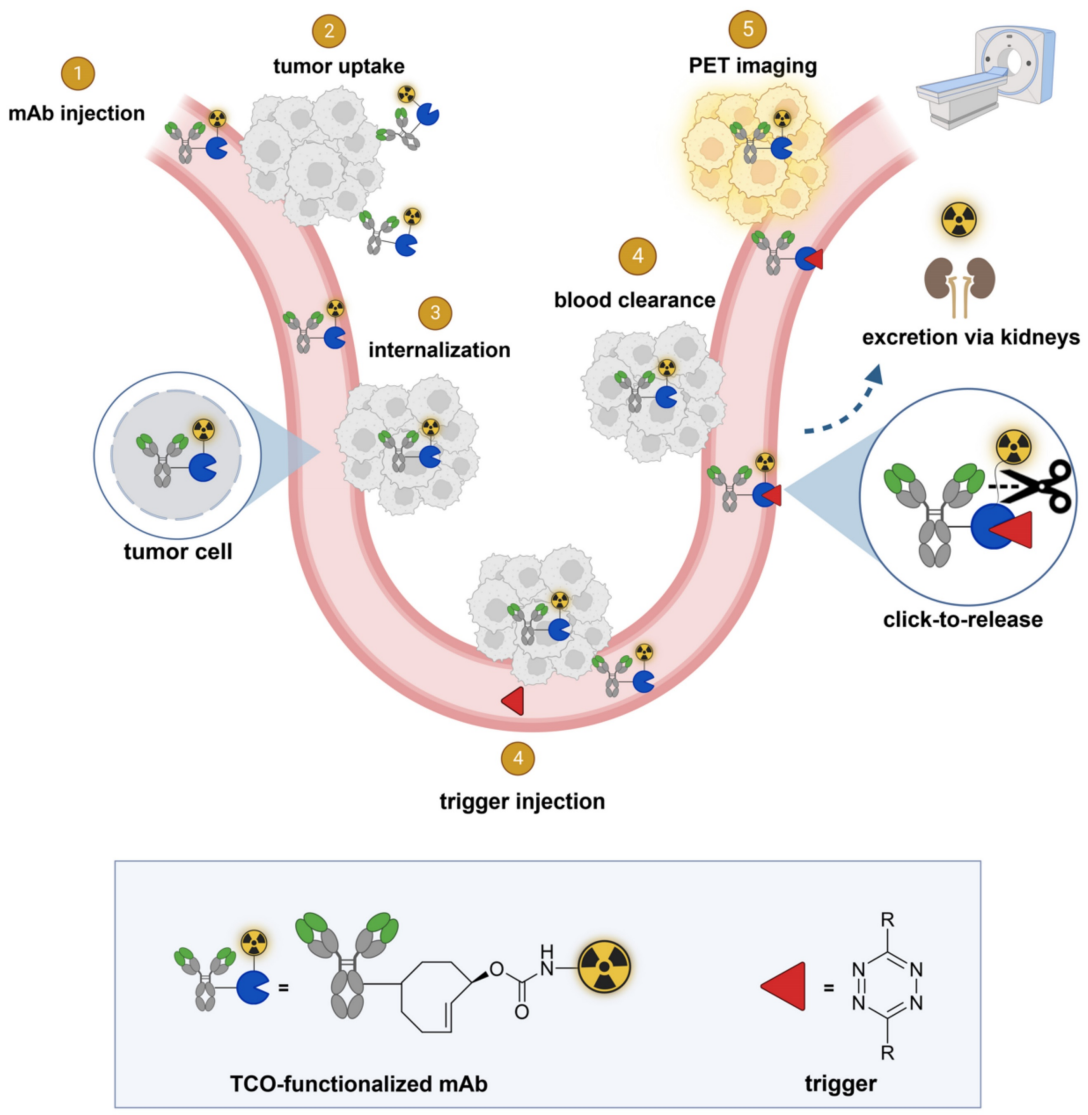
** General concept of cleavable radioimmunoimaging using click-to-release chemistry *in vivo*.** The TCO-linked and radiolabeled mAb is administered and internalizes in the tumor cells. Once enough tumor internalization has occurred, the trigger (tetrazine) is administered, which can solely react with TCO in blood and in other extracellular compartments given its non-cell-permeable nature. The result of this bioorthogonal reaction is the rapid release of the radiolabeled chelator in circulation and its efficient renal excretion, increasing the T/B ratio. Created with Biorender.com

It would be advantageous to be able to separate the radioactive moiety from the mAb in blood and other non-target tissues, after sufficient tumor uptake has occurred, allowing rapid renal clearance of the radioactivity, boosting tumor-to-background ratios. mAb-chelate conjugates cleavable by endogenous or exogenous enzymes have been developed 16-18. However, the former approach was designed to reduce the radioactivity in liver instead of in blood 16 and the latter approach was hampered by the need for multiple doses of enzyme with the risk of immunogenicity 18. We set out to develop a method where the separation of the radioactivity from the mAb occurs in blood and is induced by an external stimulus based on a bioorthogonal reaction, enabling a temporal and potentially spatial control over the cleavage (Figures 1 and 2).

We previously developed a bioorthogonal cleavage reaction (Figure 1) based on the inverse electron-demand Diels-Alder (IEDDA) reaction between a *trans*-cyclooctene (TCO) and a tetrazine 19. This IEDDA pyridazine elimination was used for on-target activation of non-internalizing chemically-cleavable antibody-drug conjugates (ADCs), wherein a TCO linker releases the drug from the allylic position upon reaction with a separately administered tetrazine in the extracellular tumor microenvironment (Figure 1) 20,21. Based on the high and selective *in vivo* reactivity combined with good stability that we observed for the chemically-cleavable ADC, we hypothesized that this reaction could be equally effective for controlled off-target deactivation and started to develop this in the context of click-cleavable radioimmunoimaging and -therapy 22.

In this approach, a mAb targeting an internalizing receptor is conjugated with a TCO-linked chelator, radiolabeled, and administered intravenously (i.v.). After sufficient tumor accumulation and internalization has occurred, a non-cell permeable tetrazine, referred to as “trigger”, is administered in a second step. The trigger reacts solely with the mAb in blood and in other extracellular domains, resulting in the release of the radiolabeled chelator, which as a small fragment clears rapidly via the kidneys while the intracellular radioactivity in the tumor is retained (Figure 2). Zhang and co-workers recently reported a similar approach using *in vivo* NO generation from the vasodilating drug glyceryl trinitrate (GTN) as the trigger for the release of the radionuclide ^131^I from a nanoparticle 23. While elegant, it is not clear whether the pharmacodynamics of the drug itself limits its applicability as a trigger molecule for the present application, and whether the NO levels are sufficiently high in plasma (i.e. in the extracellular blood compartment).

**Figure 3:**
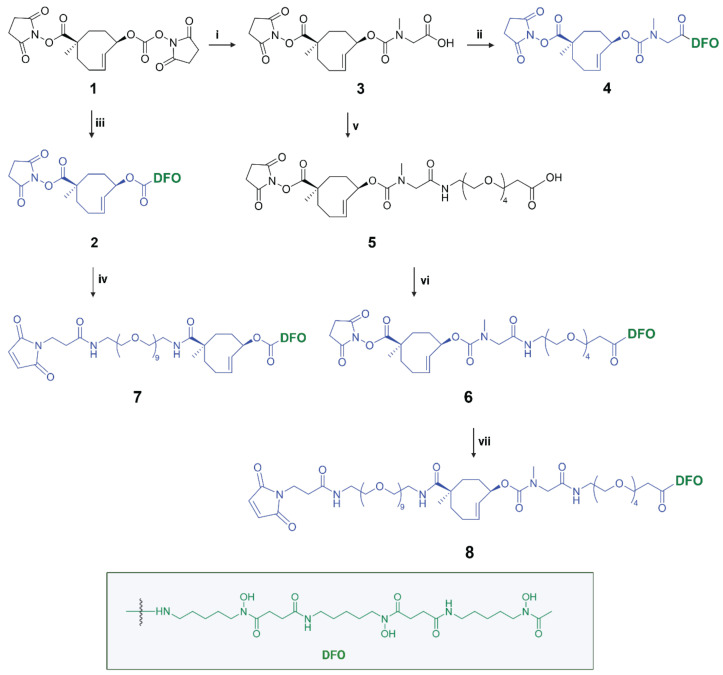
** Synthesis of chemically-cleavable linker-chelators comprising a TCO functionalized on the releasing end (allylic position) with the [^89^Zr]Zr-DFO chelator.** Reagents and conditions: (i) sarcosine, water, 10 min, RT (quantitative); (ii) PyBOP, DIPEA, deferoxamine mesylate salt, DMSO, 3h, RT (83%); (iii) deferoxamine mesylate salt, DMSO, 4 h, RT (84%); (iv) mal-amido-PEG_9_-amine, DMF, DIPEA, RT; (v) PyBOP, DIPEA, DMF, amino-PEG_4_-acid, 2 h, RT (14%); (vi) PyPOB, DIPEA, deferoxamine mesylate salt, DMSO, 4 h, RT (86%); (vii) mal-amido-PEG_9_-amine, DMF, DIPEA, 2 h, RT (11%).

Here we describe the development of click-cleavable radiolabeled trastuzumab (Tmab), which binds the internalizing HER2 receptor 24,25. ^89^Zr-labeled Tmab is used extensively to image HER2-positive cancer patients with PET 8,26,27. We synthesized a small series of TCO-deferoxamine (TCO-DFO) linker-chelator constructs comprising a conjugation moiety and PEG spacers on one or both sides of the TCO to increase hydrophilicity and to potentially modulate the performance of the click-to-release reaction (Figure 3). Following conjugation to Tmab and ^89^Zr-labeling, trigger-induced [^89^Zr]Zr-DFO release was tested *in vitro* and the most promising [^89^Zr]Zr-TCO-Tmab/trigger pair was then evaluated *in vivo*, confirming that PET imaging of cancer with full mAbs can be significantly improved using click-cleavable radioimmunoconjugates.

## Materials and Methods

Additional methods, materials, corresponding figures, and tables are provided in the Supplementary Information, including synthesis and characterization of all new compounds, cell internalization experiments, *in vitro* release experiments, *in vivo* experiments and PET imaging.

### Antibody conjugations

Tmab in chelex-treated PBS was reacted with 35 equivalents of linker-chelator constructs **2**, **4**, and **6** in PBS (pH adjusted to 8.5), to afford conjugates **Tmab-2**, **Tmab-4** and **Tmab-6**, respectively. Constructs **7** and **8** were conjugated to Tmab after partial mAb reduction with TCEP (2.3 eq) followed by incubation with 10 equivalents of the constructs in PBS pH 6.8 (in the presence of 2 mM EDTA) to afford the conjugates **Tmab-7** and **Tmab-8**, respectively 28. For all conjugates the excess of unreacted linker-chelator was removed by Size Exclusion Chromatography (SEC), with the UV lamp off, in order to obtain the final conjugates with >95% purity, as confirmed by analytical SEC. The amount of TCO per Tmab in the conjugates was measured using a tetrazine titration 21 and was found to range from 1.2 to 1.8 for **Tmab-2**, **Tmab-4** and **Tmab-6** and from 2.1 to 2.5 for **Tmab-7** and **Tmab-8**. All the conjugate solutions were stored in aliquots at -80 ºC.

### ^89^Zr labeling

The radiolabeling of all conjugates was performed following a published procedure with slight modifications 29. Zr-89 oxalate solution (ca 6MBq, 20 µL) was combined with 7 μL of 2M Na_2_CO_3_. The reaction was left at room temperature (RT) for 5 min, followed by addition of 173 μL of 0.5M HEPES (pH 7.4) and finally by the Tmab conjugate solution (40 μg) in a final volume of 200 μL with a pH between 7.2 - 7.4 After incubation at 37 ºC, the radiolabeling yield determined by iTLC varied between 80% and 95%. The radiolabeling mixtures were purified using Zeba spin desalting columns (0.5 mL, 40 kDa MW cut-off). The purity of the recovered radiolabeled conjugates was >95% as determined by iTLC, SDS-PAGE and FPLC. The stability of ^89^Zr-incorporation in the mAb conjugates was evaluated by iTLC after incubation in 50% human or mouse serum for up to 4 days at 37 ºC.

### Release experiments *in vitro*

The release experiments in PBS were carried out in triplicate mixing 1.6 μg of [^89^Zr]Zr-TCO-Tmab (0.13 MBq) with an excess of trigger (300 eq, from 1 mM solution in DMSO). Before analyzing the reaction mixture using SEC, 50 μL of a 0.6% BSA solution in PBS was added. The release experiments in plasma were performed by mixing 1.6 µg of [^89^Zr]Zr-TCO-Tmab (0.13 MBq) with an excess of each trigger (300 eq, from 1 mM solution in DMSO), while the final volume was 1:1 (plasma : PBS).

### Animal studies

For animal experiments, the guidelines set by the Nijmegen and European Animal Experiments Committee were followed and all experiments were approved by the institutional Animal Welfare Committee of the Radboud University Nijmegen. Female BALB/c nude mice (7-9-week-old, 18-22 g body weight) were used. All animals were allowed to acclimate for 1 week before the start of the experiments. Upon arrival, the mice were identified with tattoos. All experiments were blinded.

For the experiments in tumor-bearing mice, on day 0 a pellet of 17β-estradiol (0.18 mg/pellet, 60-day release) was subcutaneously (s.c.) implanted in the mice followed by injection with BT-474 cells (5 million cells / mouse in 100 μL matrigel / RPMI 1:1) in the mammary fat pad, under anesthesia. Tumor size was determined by caliper measurements in three dimensions (tumor volume = ½ × 1 × w × h) twice per week and the tumors were allowed to grow for 3-4 weeks until the volume reached 30 - 50 mm^3^, at which point the animals were randomly allocated to the various groups. At euthanasia, blood was obtained by cardiac puncture and organs and tissues of interest were harvested, blotted dry, and weighed. The sample radioactivity was measured in a gamma counter (Wizard 1480, PerkinElmer) along with standards to determine the % injected dose per gram (% ID/g) and the % injected dose per organ (% ID/organ). Stomachs, small and large intestines were not emptied before γ-counting.

### Tmab conjugate blood kinetics in mice

Tumor-free mice (n = 4) were injected i.v. with **[^89^Zr]Zr-Tmab-8** (0.5 mg/kg, ca 0.5 MBq in 100 μL saline per mouse). Blood was withdrawn via the vena saphena (ca 20 μL samples) at various time points between 1 h and 72 h post mAb injection. Four days post-mAb injection the mice were euthanized, one last blood sample was obtained via a cardiac puncture and selected organs and tissues were harvested for γ-counting. The half-lives in blood were calculated by fitting the data to bi-exponential curves.

### Tmab conjugate stability in mice

Τumor-free nude mice (n = 4) were injected i.v. with **[^89^Zr]Zr-Tmab-8** (0.5 mg/kg, ca 0.5 MBq in 100 μL saline per mouse). At selected time points (1 h, 3 h, 6 h, 24 h, 48 h, 72 h and 96 h) blood samples were withdrawn from the vena saphena and collected in vials containing heparin. After radioactivity measurement, the blood samples were diluted with 200 μL PBS and the blood cells were removed by centrifugation. The supernatant was divided in two aliquots. One aliquot of supernatant was analyzed by SEC. To the other aliquot an excess of trigger was added and the solution was incubated at 37 ºC overnight followed by SEC analysis to quantify the extent of chelate release. The % release was normalized to the value obtained at t=0 and was plotted with time. The data were then analyzed using linear regression.

### Triggered Tmab conjugate cleavage in tumor-free mice

Tumor-free mice (n = 4) were injected i.v. with **[^89^Zr]Zr-TCO-Tmab-8** (0.5 mg/kg, ca 0.5 MBq in 100 μL saline per mouse), followed 1 h later by an i.v. dose of trigger (33.4 μmol/kg in 100 μL PBS with 5% DMSO). One group of mice received one extra dose of trigger 2 h after the first trigger injection. Blood was withdrawn via the vena saphena (ca 20 μL samples) at various time points. The blood samples were weighed and were measured in a γ-counter along with standards. Mice were euthanized 24 h post-mAb injection and selected organs were harvested, weighed, and measured in the γ-counter.

### Triggered Tmab conjugate cleavage in tumor-bearing mice

Mice bearing BT-474 xenografts (n = 5) were injected i.v. with **[^89^Zr]Zr-Tmab-8** (0.5 mg/kg, ca 0.5 MBq in 100 μL saline per mouse), followed at 6 h or 24 h post-mAb injection with an i.v. dose of trigger **10** (33.4 μmol/kg, in 100 μL PBS with 5% DMSO). Mice were euthanized 4 h after the trigger dose in both cases. The organs were harvested, weighed, and measured in the γ-counter.

### PET studies

Mice bearing BT-474 xenografts (n = 4) were injected i.v. with **[^89^Zr]Zr-Tmab-8** (0.5 mg/kg, ca 5 MBq in 100 μL saline per mouse). After 5 h mice were imaged for 15 min under anaesthesia (2-3% isoflurane in air). One hour after recovery from anaesthesia the mice received a dose of **10** i.v. (33.4 μmol/kg in 100 μL PBS with 5% DMSO) and, 4 hours later, the mice were anaesthetized again and imaged for 30 min. Inveon Acquisition Workspace software (version 1.5, Siemens Preclinical Solution, Erlangen, Germany) was used for PET scans reconstruction with an algorithm with shifted Poisson distribution and the following settings: matrix 256 × 256 × 161, pixel size 0.4 × 0.4 × 0.8 mm, with a corresponding beta of 0.05 mm.

### Data analysis

All data are presented as the mean ± standard deviation (SD). Curve fitting, linear regressions and area-under-the-curve calculations were performed with GraphPad Prism (v 9). Statistical analysis was performed using the Student's t-test (two-tailed) with Welch's correction and (un)paired t-test using GraphPad Prism (v 9). Statistical significance was set at p < 0.05.

## Results and Discussion

### Design and synthesis of TCO-DFO linker-chelator conjugates

Detailed information for the synthesis of the TCO linker-chelator constructs is provided in the Supplementary Information. In this study we synthesized a series of chemically-cleavable linker-chelator conjugates (compounds **2, 4, 6, 7** and** 8**) containing a TCO functionalized on the releasing end (allylic position) with DFO for ^89^Zr-labeling (Figure 3). All compounds were obtained starting from the bis-NHS-functionalized TCO (**1**) that was previously used for the development of chemically-cleavable ADCs 20, 21. In linkers **6-8**, PEG spacers were introduced before the TCO, or between the TCO and the DFO chelator, or both (Figure 3) to increase the hydrophilicity of the linkers and to potentially influence the efficiency of the click-to-release reaction with the trigger. The N-methyl group on the carbamate in **4**, **6**, **7** and **8** was added to prevent a potential intramolecular side-reaction, which was found by Carlson *et al*. to produce a non-releasing species 30.

All constructs were conjugated to Tmab either via NHS chemistry or maleimide chemistry producing **Tmab-2**, **Tmab-4**, **Tmab-6**, **Tmab-7** and **Tmab-8,** which were radiolabeled with Zr-89 to afford **[^89^Zr]Zr-Tmab-2**, **[^89^Zr]Zr-Tmab-4**,** [^89^Zr]Zr-Tmab-6**, **[^89^Zr]Zr-Tmab-7** and **[^89^Zr]Zr-Tmab-8**, respectively, with high radiochemical purity (Figure S2) and long *in vitro* stability in both human and mouse serum (Figure S5).

### Release experiments *in vitro* and in circulation in mice

**Figure 4:**
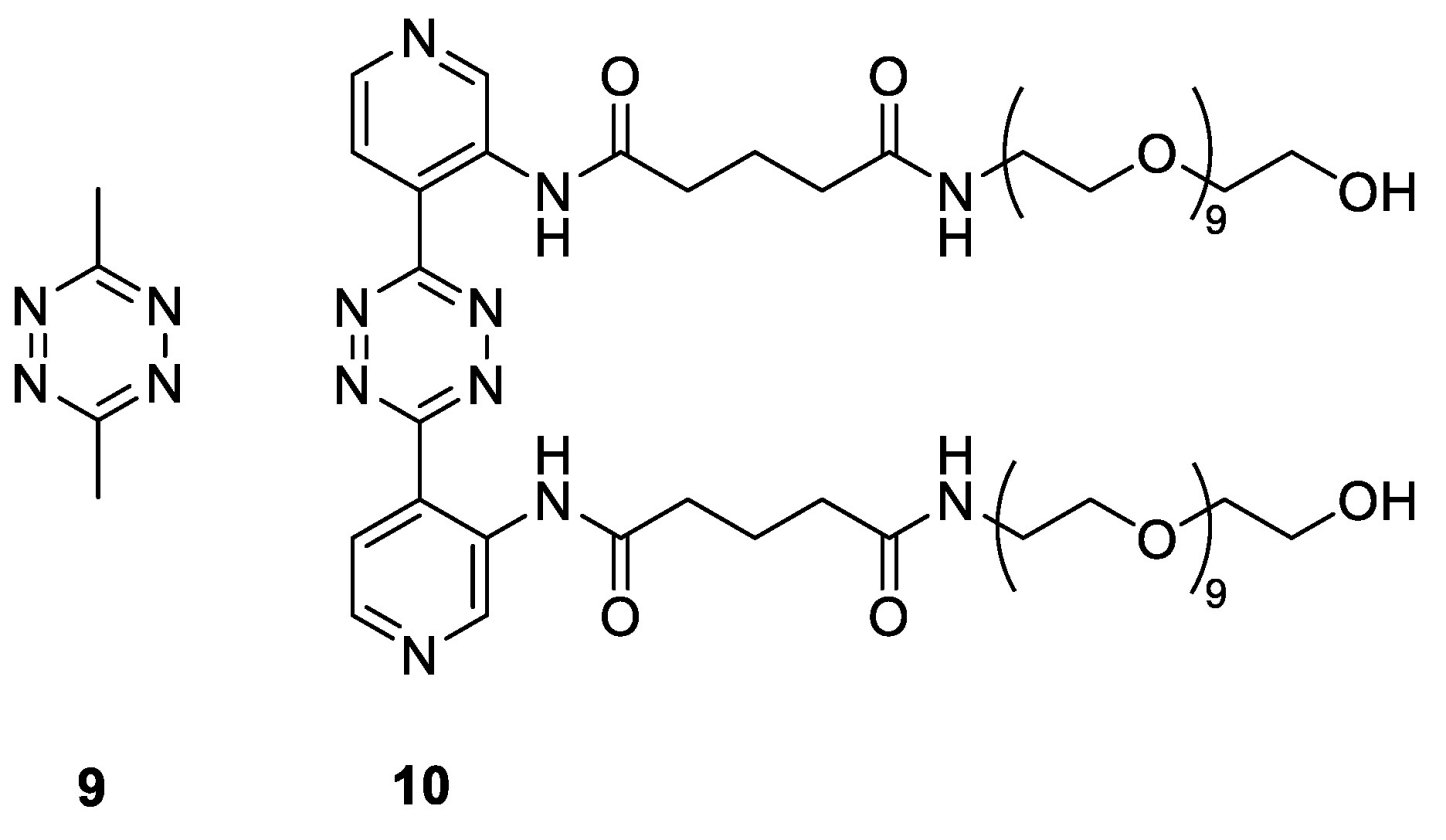
Triggers used in this study.

The IEDDA pyridazine elimination reaction requires the use of tetrazines that upon reaction with the TCO linker efficiently afford the releasing 1,4-dihydropyridazine intermediate from the initially formed 4,5-dihydropyridazine cycloaddition product 19. The highly reactive and biocompatible 3,6-bispyridyl-1,2,4,5-tetrazine motif, the tetrazine of choice for many *in vivo* applications such as pretargeted radioimmunotherapy 31, unfortunately does not afford an efficiently releasing 1,4-dihydropyridazine intermediate with TCO constructs derived from **1** 19. The 3,6-bismethyl-1,2,4,5-tetrazine (**9**, Figure 4) gives high release of TCO-conjugated payloads *in vitro* 19, however, its low reactivity and fast pharmacokinetics makes it less suitable for *in vivo* applications 21. To harness the high reactivity of 3,6-bispyridyl tetrazine for improved *in vivo* efficacy of click-to-release approaches, we recently developed tetrazines with ortho-functionalized pyridyl substituents, such as the amide-substituted **10** (Figure 4), designed to promote the formation of the releasing 1,4-dihydropyridazine tautomer 32. In contrast with the ca. 10% release achieved with the parent bispyridine-tetrazine 19, this compound yielded 80% *in vitro* antibody-drug conjugate (ADC) cleavage within 2 h increasing to near quantitative overnight, and exhibited efficient *in vivo* ADC cleavage as well.

**Figure 5:**
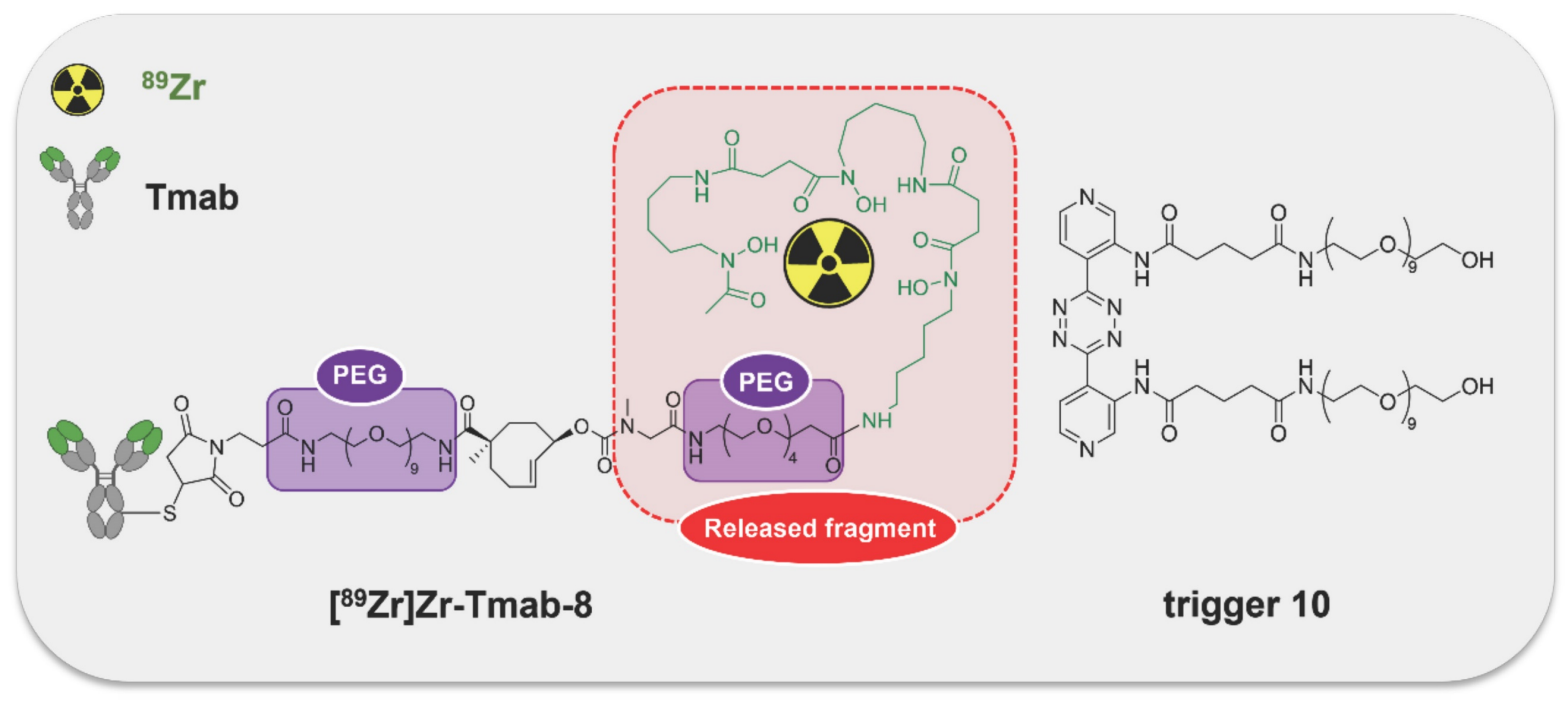
** The [^89^Zr]Zr-TCO-Tmab and trigger pair used in the *in vivo* experiments.** Structure of **[^89^Zr]Zr-Tmab-8**, comprising PEG spacers before and after the TCO and structure of trigger **10**.

Compound **10** was evaluated as trigger for the cleavage of the [^89^Zr]Zr-TCO-Tmab conjugates in PBS for 24 h and compared with reference compound **9** (Table 1, Figure S1). While in general the release achieved with **10** was somewhat lower and the linker variations did not lead to significant differences, the highest release found with **[^89^Zr]Zr-Tmab-8** with 78% in PBS increased to 83% in 50% mouse plasma (Figure S3 and S4), which is close to what is maximally achievable with **9** 19-21. Therefore, the combination of **10** with **[^89^Zr]Zr-Tmab-8** was selected for *in vivo* evaluation (Figure 5).

**Figure 6:**
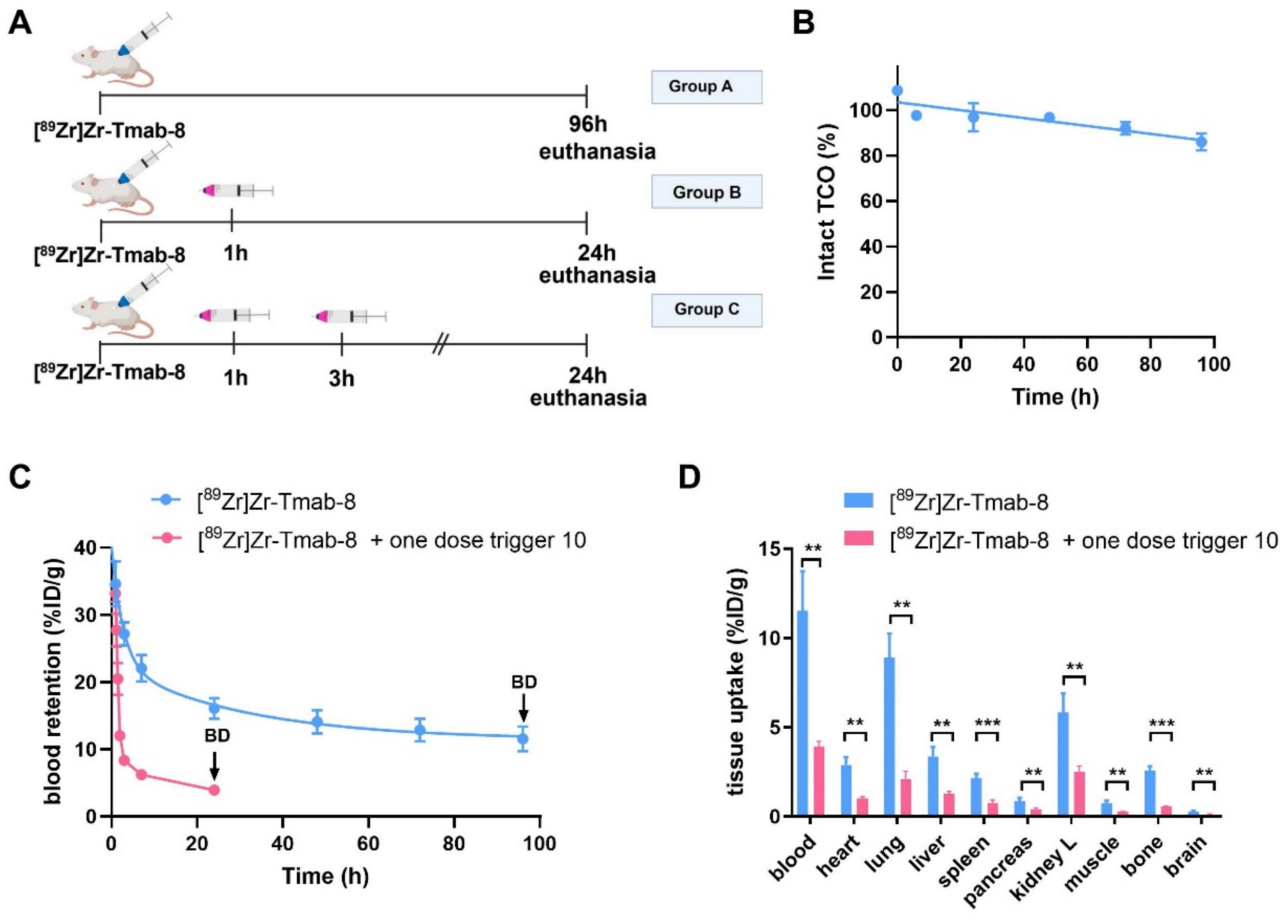
** Evaluation of [^89^Zr]Zr-Tmab-8 and trigger 10 in tumor-free mice. (A)** Experimental scheme of *in vivo* studies, where tumor-free mice received **[^89^Zr]Zr-Tmab-8** alone (Group A), mAb followed by one dose of trigger **10** (Group B) or two doses of trigger **10** (Group C). **(B)** Normalized *in vivo* TCO linker stability in tumor-free mice. **(C)** [^89^Zr]Zr blood clearance profile of **[^89^Zr]Zr-Tmab-8** alone and with trigger **10** 1 h post-**[^89^Zr]Zr-Tmab-8** administration. The data represent the mean % ± SD (n = 4),** (D)** Biodistribution (BD) at respectively 96 h and 24 h post-mAb injection of mice treated with **[^89^Zr]Zr-Tmab-8** alone (blue) and in combination with one dose of trigger **10** 1 h post-**[^89^Zr]Zr-Tmab-8** (pink). Statistical analysis was performed using the unpaired Student's t-test (**p < 0.01 and ***p < 0.001).

The blood clearance of** [^89^Zr]Zr-Tmab-8** (0.5 mg/kg) alone in tumor-free mice was slow and bi-phasic (1.86 h t_1/2,α_ and 21.50 h t_1/2,β_), and matches the typical blood clearance of ^89^Zr-labeled Tmab, suggesting that the TCO-modification does not change the *in vivo* behavior of the radioimmunoconjugate (Figure 6A, Group A; Figure 6C).

Regarding the triggered release, previous studies have shown that the radioactive moiety [^89^Zr]Zr-DFO is eliminated from the blood via the kidneys within 20-60 min and we assumed that the released fragment (Figure 5) from circulating **[^89^Zr]Zr-Tmab-8** would exhibit similar pharmacokinetics 33,34. We were therefore pleased to observe that in mice that received **[^89^Zr]Zr-Tmab-8** followed by one dose of trigger **10** (33.4 μmol/kg) (Figure 6A, Group B; Figure 6C) the radioactivity in blood was 4-fold lower compared to the mice that did not receive the trigger, with 3.93 ± 0.29 %ID/g and 16.08 ± 1.51 %ID/g (p = 0.0004) at 24 h post-mAb injection, respectively. This reduction, which also afforded greatly reduced radioactivity levels across most tissues upon biodistribution (Figure 6D), corresponds well with the *in vitro* cleavage yield but to check if there was any unreacted TCO left in blood, a second dose of trigger **10** was administered 2 h after the first dose (Figure 6A, Group C). No significant decrease of the radioactivity in blood (3.61 ± 0.20 %ID/g, p = 0.1291) was observed after the second trigger dose, confirming that all TCO in blood had already reacted with the first trigger dose (Table S1, Figure S7).

Previous studies have determined that TCO can isomerize to the unreactive *cis*-isomer in the presence of serum proteins 35. To assess the deactivation rate of the TCO as Tmab-DFO linker we collected blood samples from healthy mice that received **[^89^Zr]Zr-Tmab-8** and reacted those with an excess of **10** *ex vivo* followed by SEC analysis to quantify the fragment release percentage as a measure of TCO content. The *in vivo* TCO deactivation half-life of the **[^89^Zr]Zr-Tmab-8** was calculated to be 16.5 days (Figure 6B), which is 3-fold higher than what we have found for TCO-linked ADCs 20,21, allowing long intervals between mAb and trigger if needed.

### Release experiments in cell culture and in tumor-bearing mice

The presented approach centers on the use of a non-cell-permeable trigger to release the radioactive fragment from the mAb in blood at a time point where enough radiolabeled mAb has internalized into cancer cells, out of reach of the trigger. To get an indication of the required interval between mAb and trigger injection we first investigated the binding and internalization of **[^89^Zr]Zr-Tmab-8** in HER2-positive BT-474 cancer cells, which we found to be fast. The total mAb cell binding increased from 74.2% to 91.3% from 6 h to 24 h, with only 17.4% and 9.9% of the radioactivity surface-bound at these two time points (Figure S6). These findings align with published data showing that indeed some Tmab remains on the cell surface 36, although in our studies this fraction is much smaller.

In mice bearing BT-474 xenografts Holland *et al.* showed increasing [^89^Zr]Zr-trastuzumab uptake in tumors from 1 h to 72 h post-mAb injection with T/B ratios > 1 only at 24 h post-mAb injection and later, due to long mAb circulation 37. In patients this translates into the need to wait 4-5 days after [^89^Zr]Zr-trastuzumab administration before PET images with optimal contrast can be acquired 8, while shorter intervals would be preferred if possible. Based on our *in vitro* results, we speculated that significant Tmab internalization *in vivo* could already be achieved 6 h post-mAb injection therefore enabling T/B ratios > 1 at times shorter than 24 h post-mAb using our click-to-release approach.

**Figure 7:**
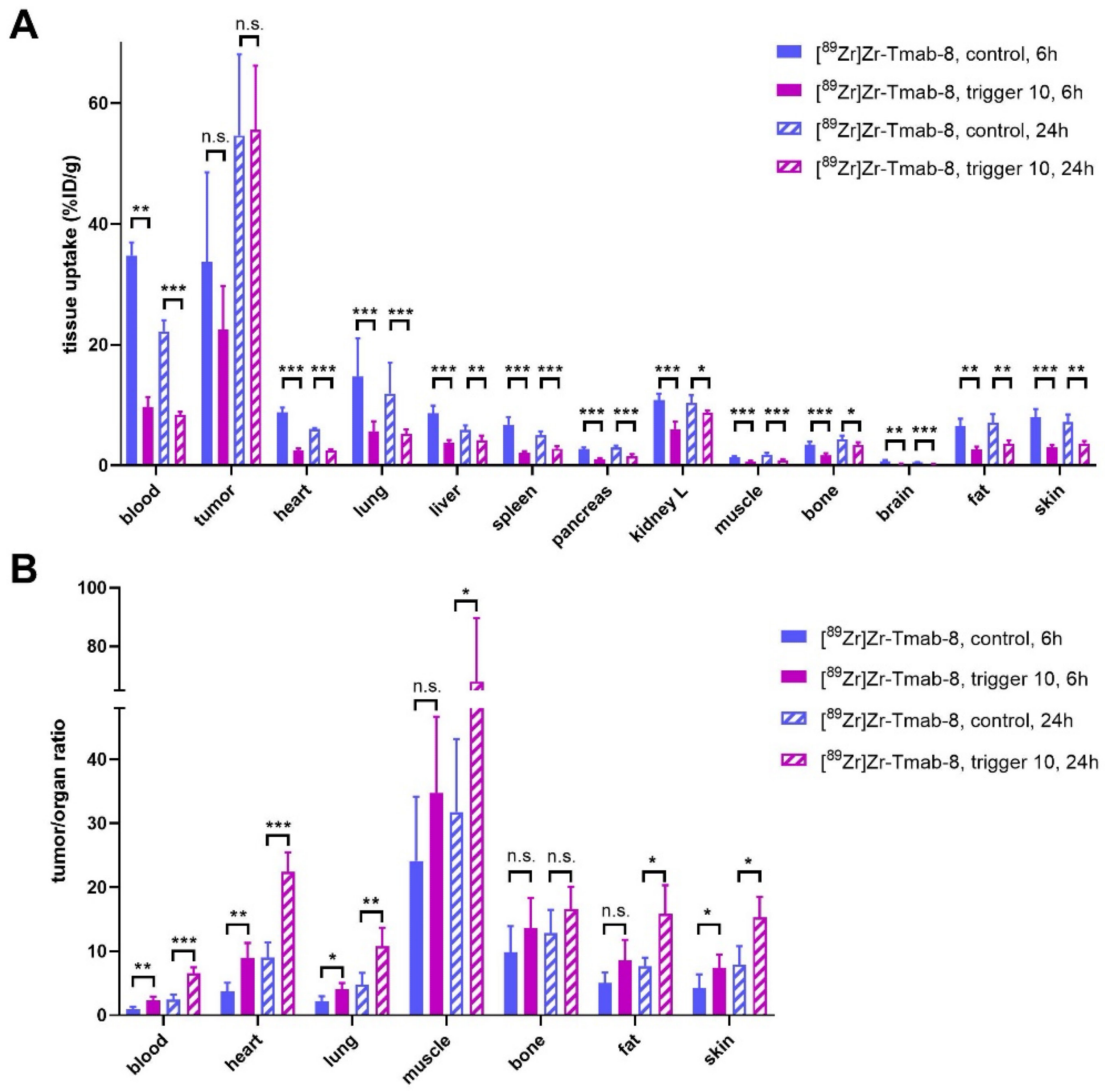
** Evaluation of [^89^Zr]Zr-Tmab-8 and trigger 10 in mice bearing BT-474 xenografts at two different time points. (A)** Biodistribution (4 h post-trigger administration) of mice bearing BT-474 xenografts receiving **[^89^Zr]Zr-Tmab-8** alone or followed by trigger **10** at 6 h or 24 h post-mAb **(B)** Tumor-organ ratios from the mice in panel A The data represent the mean % ± SD (n = 5). Statistical analysis was performed using the unpaired Student's t-test, *p < 0.05, **p < 0.01, ***p < 0.001 and n.s.= not significant (A: tumor, 6 h: p = 0.1793; 24 h: p = 0.9042; B: tumor-muscle, 6 h: p = 0.1682; tumor-bone, 6 h: p = 0.2119; 24 h: p = 0.1219; tumor-fat, 6 h: p = 0.0719).

Therefore, we set out to investigate the release of **[^89^Zr]Zr-Tmab-8** by trigger** 10** in mice bearing orthotopic BT-474 xenografts, with 6 h and 24 h interval between mAb and trigger administration (biodistribution at 4 h post-trigger; Figure 7, Table S2 and S3). When the trigger was administered 6 h post-mAb, as expected, a minor fraction of the tumor-bound radioactivity appeared to wash out from the tumor (p = 0.1793) due to incomplete Tmab internalization at this timepoint. However, the T/B ratio reached 2.3 ± 0.6, which is 2.3-fold higher than the T/B of 1.0 ± 0.4 for the control group (p = 0.0057) (Figure 7B). The 24 h interval afforded a T/B ratio of 6.6 ± 0.9, which is 2.6-fold higher than the T/B of 2.5 ± 0.7 for the control group (p < 0.0001) (Figure 7B). The slightly higher T/B ratio improvement at 24 h time point is consistent with the slightly higher internalization found *in vitro* in cells at that time and is due to the increased retention of radioactivity in the tumor at 24 h in the group receiving the trigger (Figure S6).

Furthermore an improved tumor-organ ratio was observed for blood-rich organs, such as the liver (from 3.8 ± 1.1 to 6.0 ± 1.9, p = 0.0673 for 6 h and from 9.3 ± 2.2 to 13.6 ± 3.4, p = 0.0494 for 24 h) and lungs (from 2.2 ± 0.8 to 4.0 ± 1.0, p = 0.0148, for 6 h and from 4.8 ± 1.8 to 10.8 ± 2.9, p = 0.0066, for 24 h), after the trigger injection at both time points (Table S3 and Figure 7B). The complete intratumor retention of the radioactivity already at 24 h post-mAb injection combined with the pronounced reduction in blood is promising for future clinical applications, as the Tmab blood clearance will be much slower in humans 8 while the tumor cell internalization rate is likely to be similar, potentially affording a larger reduction of the radiation dose in blood.

### Small animal PET imaging

**Figure 8:**
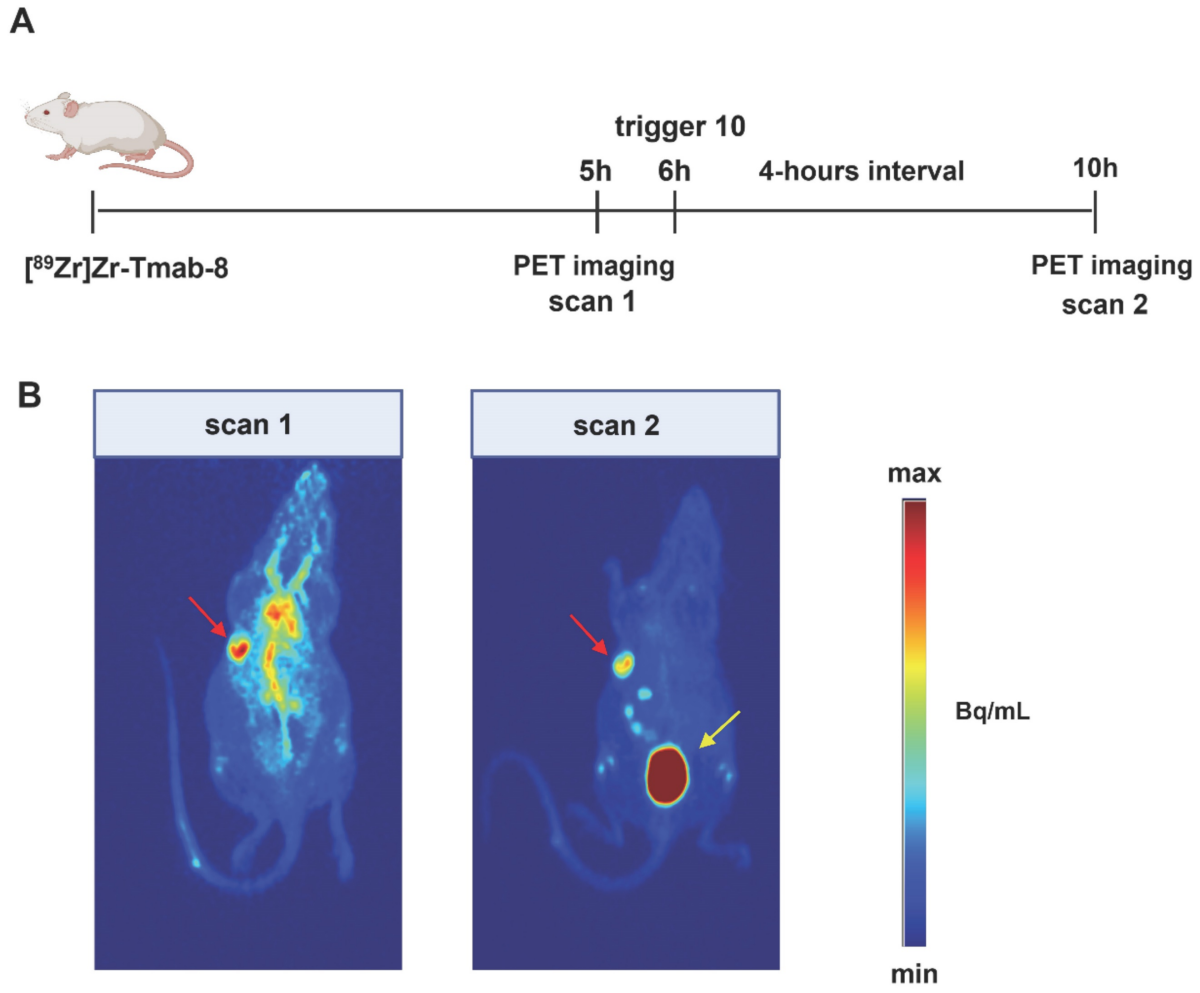
** PET imaging studies in tumor-bearing mice. (A)** Experimental scheme of the PET imaging studies. Mice bearing HER2-positive BT-474 xenografts (n = 4) received **[^89^Zr]Zr-Tmab-8** and 5 h later were imaged under anesthesia (scan 1). One hour later the mice received trigger **10** and 4 h post-trigger **10** administration were imaged again under anesthesia (scan 2) **(B)** Representative PET maximum intensity projections from the same mouse, before trigger administration (scan 1) and after trigger administration (scan 2). The images are scaled to the same min and max values. Red arrows indicate the tumor site and yellow arrow indicates the bladder.

Given the comparable T/B ratio improvement with the 6 h and 24 h interval we sought to investigate whether the tumor-nontumor ratio improvement at 6 h would allow effective same-day imaging of a radiolabeled mAb. To this end, mice received **[^89^Zr]Zr-Tmab-8** followed by **10** 6 h later, with PET scans taken 1 h prior and 4 h post-trigger administration (Figure 8, Table 2, Figure S8). The results clearly indicate that before the trigger administration (scan 1) there are high radioactivity levels in blood and in blood-rich organs (tumor-heart ratio 0.9 ± 0.1), which increases the background and hinders the visibility of the tumor (red arrow, scan 1 in Figure 8B). To our delight, upon triggered chelate release the background radioactivity was largely reduced, greatly improving the tumor image (tumor-heart ratio 2.4 ± 0.7, p = 0.0229, scan 2 in Figure 8B). The PET images obtained are in accordance with the biodistribution data, which afforded a T/B ratio of 2.3 ± 0.6 (p = 0.0057) and they show that the clearance of the released small fragment occurs mainly via the kidneys into the bladder (yellow arrow, scan 2 in Figure 8B). A small amount of radioactive fragment was also found to clear via the intestines as visible in the PET images (Figure 8B, scan 2, Figure S8, D, E and F) and confirmed by the biodistribution data (Table S2). This was also found by Bhatt et al. for [^89^Zr]Zr-DFO 33. In a pretargeting study using TCO-functionalized Tmab and a ^18^F-labeled tetrazine tracer, Lewis *c.s.* reported lower T/B ratios (0.57 ± 0.04), likely due to the low amount of non-internalized TCO-trastuzumab available for the tetrazine reaction at 48 h 38. In contrast, the click-to-release approach presented herein uses the fast mAb internalization to its advantage.

## Conclusion

We have demonstrated the successful use of click-to-release chemistry for the efficient and temporally controlled cleavage of a radioactive chelator from an antibody *in vivo*. This approach provided excellent off-target deactivation of the radiolabeled mAb with fast renal clearance of the cleaved radioactive fragment while largely retaining the on-target activity in the tumor both when using the trigger at 6 h and 24 h post-mAb administration. Future work will focus on further increasing the *in vivo* cleavage yields to maximize the benefit. We believe that the presented click-cleavable radioimmunoimaging approach may allow for lower whole-body radiation doses in the clinic, potentially same day imaging, and improved image quality. While the present report demonstrates the benefits in the context of a cancer cell-internalized mAb we expect the approach to be equally promising for imaging of mAbs designed to cross the blood brain barrier, allowing selective radioactive background reduction in cerebral blood. We think the approach may also find application in increasing target-nontarget ratios of smaller targeting agents. Finally, we believe it may be highly beneficial for radioimmunotherapy by reducing the bone marrow dose, thereby enabling increased tumor doses.

## Supplementary Material

Supplementary figures and tables, information.

## Figures and Tables

**Table 1:** ** Triggered release of radioactive fragment from [^89^Zr]Zr-TCO-Tmab conjugates in PBS.** ^89^Zr-conjugates were mixed in PBS with an excess of trigger (300 eq) and incubated at 37 ºC for 24 h. Samples were analyzed using SEC. The data represent the mean % ± SD (n = 3).

^89^Zr-labeled Tmab	Release induced by triggers (mean% ± SD)		
	9	10	
[^89^Zr]Zr-Tmab-2	80.4 ± 0.6	69.3 ± 1.7	
[^89^Zr]Zr-Tmab-4	88.5 ± 0.3	75.1 ± 0.8	
[^89^Zr]Zr-Tmab-6	86.9 ± 0.1	68.2 ± 4.2	
[^89^Zr]Zr-Tmab-7	63.2 ± 2.3	59.4 ± 9.8	
[^89^Zr]Zr-Tmab-8	89.5 ± 0.5	78.1 ± 0.6	

**Table 2:** ** PET quantification.** Mice (n = 4) bearing BT-474 xenografts were injected with **[^89^Zr]Zr-Tmab-8** (ca 0.5 mg/kg, ca 5 MBq) and 5 h later were imaged to obtain scan 1. After 1 h they received a dose of trigger **10** and 4 h post-trigger injection they were imaged again to obtain scan 2. PET scans were reconstructed and the tumor-muscle and tumor-heart ratios before and after trigger administration were obtained. Data were compared using paired t-test for tumor-heart ratio (p = 0.0229) and tumor-muscle ratio (p = 0.1248). The data represent the mean % ± SD (n = 4).

PET scans	Tumor-muscle ratio
Scan 1	8.4 ± 2.3
Scan 2	20.2 ± 8.8
	**Tumor-heart ratio**
Scan 1	0.9 ± 0.1
Scan 2	2.4 ± 0.7
